# Recanalization Therapy for Acute Ischemic Stroke with Large Vessel Occlusion: Where We Are and What Comes Next?

**DOI:** 10.1007/s12975-020-00879-w

**Published:** 2021-01-06

**Authors:** Mohammad Shafie, Wengui Yu

**Affiliations:** Department of Neurology, University of California, Irvine, 200 S. Manchester Ave., Suite 206, CA 92868 Orange, USA

**Keywords:** Acute ischemic stroke (AIS), Endovascular thrombectomy (EVT), Ischemic penumbra, Perfusion mismatch, Symptomatic intracerebral hemorrhage (sICH), Therapeutic time window, Clinical imaging mismatch

## Abstract

In the past 5 years, the success of multiple randomized controlled trials of recanalization therapy with endovascular thrombectomy has transformed the treatment of acute ischemic stroke with large vessel occlusion. The evidence from these trials has now established endovascular thrombectomy as standard of care. This review will discuss the chronological evolution of large vessel occlusion treatment from early medical therapy with tissue plasminogen activator to the latest mechanical thrombectomy. Additionally, it will highlight the potential areas in endovascular thrombectomy for acute ischemic stroke open to exploration and further progress in the next decade.

## Introduction

Acute ischemic stroke (AIS) persists as one of the leading causes of long-term disability and mortality both in the United States and globally despite significant advances in stroke care in the past three decades [1]. Large vessel occlusion (LVO) is determined as the underlying etiology in approximately 30–40% of ischemic strokes [2, 3], contributing to 60% of post-stroke dependence and death at 90 days and 90% of post-stroke mortality at 6 months [4]. The first line standard of care for patients with AIS within 4.5 h of symptom onset remains intravenous thrombolysis [5, 6]. However, this first line treatment has low utilization rate owing to its narrow therapeutic time window and low recanalization rate for LVO [7].

From 2015 to 2016, publication of six successful randomized clinical trials of endovascular thrombectomy (EVT) for patients with large vessel occlusion in the anterior circulation revolutionized ischemic stroke care [8–13]. In the ensuing 3 years, two additional trials showed the benefit of EVT up to 24 h from last seen well in selected patients with LVO and shifted the treatment paradigm from purely time-based to tissue-based therapy [14, 15]. Despite their resounding success, up to 60% of subjects in the treatment arms of all the recent EVT trials did not achieve functional independence [8–15]. Therefore, there remains a vast prospect for further advancements in AIS treatments, in particular with EVT, to expand the substantial clinical benefit to a larger patient population. To this end, this review encompasses a comprehensive chronological assessment of the trials and tribulations which led to the development of the evidence supporting the current practice of EVT for AIS treatment and identifies the potential areas for further improvement and deployment of this proven therapy to all whom may benefit.

## Evolution of Endovascular Reperfusion Therapies

### From Systemic Intravenous to Local Intra-arterial Thrombolysis

The narrow therapeutic time window and low recanalization rate of systemic thrombolysis with intravenous (IV) recombinant tissue plasminogen activator (r-tPA) in LVOs led the impetus for the development of endovascular reperfusion therapies [7]. To this end, the (PROACT-II) study remains as the only positive trial to demonstrate the clinical efficacy of local intra-arterial (IA) thrombolysis with recombinant pro-urokinase (r-proUK) in patients with AIS patients of less than 6-h duration caused by a proximal middle cerebral artery (MCA) M1 segment occlusion [16]. The PROACT-II trial prohibited any mechanical manipulation of the clot and showed that 40% of r-proUK and 25% of control patients had an improved neurologic outcome as measured by a modified Rankin score (mRS) ≤ 2 at 90 days after stroke onset (OR = 2.13; 95% CI, 1.02–4.42; *p* = 0.04) [16]. Despite the encouraging results of PROACT-II, the US FDA did not approve IA treatment of AIS with r-proUK based on the small sample size, marginal significance (*p* = 0.043), and approval for IV tPA treatment. In the era of recent EVT trials, the role of IA thrombolysis remains uncertain. Observational studies of IA r-tPA as either adjuvant or rescue therapy after failed thrombectomy have shown encouraging results with acceptable safety profile and improved reperfusion rates [17, 18]. The American Heart Association/American Stroke Association (AHA/ASA) guidelines continue to recommend IA thrombolysis in appropriately selected AIS patients within 6 h provided they were not candidates for IV r-tPA (Class I, Level of Evidence: B) [5].

### Mechanical Thrombectomy

Coil retrievers, the first class of mechanical thrombectomy (MT) devices approved by the US FDA, were designed as helical nitinol (a flexible nickel titanium alloy) coils, similar to a corkscrew, to entrap the thrombus and extrude it via the guide catheter [19].

Coil retrievers were approved based on the MERCI and Multi-MERCI trials [20, 21]. The Multi-MERCI trial is more relevant to current practice, as it tested a later generation of coil retriever devices subsequently used in EVT trials and enrolled both patients who were ineligible for and who failed IV r-tPA [21]. The Multi-MERCI trial, a single-arm, multicenter study that enrolled 177 patients, demonstrated partial or complete recanalization in 55% of patients with the coil retrievers alone and 69% with rescue use of additional endovascular therapies. This recanalization rate substantially exceeded the historical comparator (the heparin arm of the PROACT 2 trial, with an 18% partial recanalization rate), indicating technical efficacy. Successful recanalization was associated with higher 90-day independent neurologic outcome of mRS≤ 2 (49% vs 10%, *p* < 0.001) and with a lower mortality rate (25% vs 52%, *p* < 0.001) [21].

#### Suction Thrombectomy

Concurrent to the development of coil retrievers, aspiration devices utilizing vacuum aspiration to remove a target occlusive thrombus in AIS were being developed [19]. While manual aspiration of target thrombi can be performed through any microcatheter, progress in developing suction thrombectomy devices required a technical solution to the problem of clogging of aspiration tips. This obstacle was overcome by adding an in-bore separator wire with a bulbous tip inside the bore which the operator could continually advance and retract, disrupting the attached thrombus and pulling it ahead of the catheter [19].

The Penumbra suction thrombectomy system (Penumbra Inc. US) was cleared based on results from a prospective single-arm multicenter trial that tested the safety and efficacy of the device in 125 patients [22]. Partial or better recanalization was reported in 82% and complete recanalization in 23% of patients, the latter value equivalent to that attained with coil retrievers in MERCI and Multi-MERCI. Independent neurologic outcome tended to be more frequent with successful compared with unsuccessful recanalization (29% vs 9%, *p* = 0.06) [22]. The aspiration thrombectomy trial, THERAPY, comparing aspiration thrombectomy plus IV tPA with IV r-tPA alone, however, was stopped early (108 of a planned 692 patients) because of external evidence of the added benefit of EVT to IV r-tPA [23]. The primary efficacy outcome of functional independence (90-day mRS ≤ 2) did not differ (38% vs 30%; OR:1.4; 95% CI, 0.6–3.3; *p* = 0.52). The small numbers make these results difficult to interpret, but there was no suggestion of harm [23].

#### Randomized Controlled Trials of First-Generation Mechanical Thrombectomy Devices

The clinical utility of endovascular approaches with IA thrombolysis and then first-generation MT devices was tested in three randomized clinical trials including the IMS III [24], MR RESCUE [25], and SYNTHESIS Expansion [26]. The IMS III trial was designed to determine whether combined approach of EVT after the administration of IV r-tPA for patients with moderate-to-severe acute ischemic stroke was more effective than IV r-tPA alone [24]. The SYNTHESIS Expansion trial was designed to investigate whether endovascular treatment, including the options of a mechanical device and IA r-tPA, was more effective than IV r-tPA alone [26]. The MR RESCUE trial tested the hypothesis that a favorable penumbral pattern on imaging could identify AIS patients more likely to benefit from EVT (with Merci Retriever and/or Penumbra System) than standard medical treatment [25]. Unfortunately, all 3 trials failed to show significant clinical benefit of EVT over standard medical therapy. No mandatory requirement for vascular imaging to screen for LVO [24, 25], nascent devices [24–26], and slow enrollment [25] may be the major limitations of these studies [27]. However, a post hoc analysis of data from IMS III showed a significant outcome benefit of EVT in the subgroup of patients with proven LVO [28].

#### Development of the Second-Generation Mechanical Thrombectomy Devices

Stent retrievers were originally designed for the purpose of stent-assisted coiling and for retracting errant coils dislodged during cerebral endovascular procedures [29]. However, in continued pursuit of higher recanalization rates, a few centers resorted to these devices to extract naturally occurring thrombi with unexpected success which ultimately led to the development of the stent retrievers in ischemic stroke endovascular reperfusion therapy [30–32]. The stent retriever technology is based on self-expanding stents with multiple crisscrossing struts to ensure capture of the thrombus within the stent wall. These devices are fully deployed across the thrombus with the help of a microcatheter and subsequently after capture of the thrombus through the stent struts; the then-unfolded stent plus the thrombus are retrieved, allowing for restoration of flow in the vessel.

The evidence for better reperfusion and good neurological outcomes with stent retrievers compared with the first-generation Merci Retrieval System primarily stems from the SWIFT and TREVO 2 phase 2 trials [33, 34]. In the SWIFT trial, a parallel-group, non-inferiority trial, 113 eligible AIS patients were randomized to undergo EVT either by the Solitaire stent retriever (*n* = 58) or the Merci coil retriever (*n* = 55) device. The primary efficacy outcome of successful recanalization without symptomatic intracerebral hemorrhage (sICH) was more likely to be achieved in the Solitaire than the Merci group (64% vs 24%; *p* = 0.0001). Furthermore, at 90 days, the Solitaire group was more likely to achieve a good neurological outcome (mRS ≤ 2) (58% vs 33%; *p* = 0.02) and a lower mortality rate (17% vs 38%; *p* = 0.02) than the Merci group [33].

Similarly, in the TREVO 2 trial, 178 eligible AIS patients were randomized to undergo EVT with either the Trevo Pro stent retriever system (Stryker Neurovascular, Kalamazoo, MI) (*n* = 88) or the Merci coil retriever (*n* = 90). The primary outcome of successful recanalization was more likely to be achieved in the Trevo Pro than the Merci group (86% vs 60%; *p* < 0.0001). The Trevo Pro group was also more likely to achieve a good 90-day neurologic outcome (mRS ≤ 2; *p* = 0.013) without a difference in 90-day mortality rates (*p* = 0.1845) than the Merci group [34].

#### Phase 3 Trials of the Second-Generation Mechanical Thrombectomy Devices

Lessons from the failed IMS III, SYNTHESIS Expansion, and MR RESCUE trials, as well as the success of SWIFT and TREVO 2 trials, led to the design of several studies with more stringent selection criteria utilizing the next-generation stent retriever thrombectomy devices [24–26, 33, 34]. Subsequently in 2015 and 2016, six randomized controlled trials indisputably established the benefits of using EVT on the clinical outcome of AIS patients compared with those receiving only standard medical care [8–13]. The trials MR CLEAN, EXTEND-IA, and SWIFT PRIME proved the benefit of EVT within the first 6 h of symptom onset in patients with anterior circulation stroke [8, 9, 12]. The THRACE trial added further evidence for thrombectomy up to 5 h from symptom onset [13]. Finally, the ESCAPE and REVASCAT trials proved the benefit of EVT up to 8 h from symptom onset in anterior circulation stroke [10, 11]. The main features, key inclusion/exclusion criteria, main imaging modalities, and the thrombectomy devices used in the landmark studies are summarized in Table 1. All trials enrolled patients with severe neurologic deficits and good pre-stroke functional status and patients in both arms received IV r-tPA as a bridge to EVT when eligible. In a meta-analysis of pooled individual patient data from MR CLEAN, ESCAPE, REVASCAT, SWIFT PRIME, and EXTEND-IA, the number needed to treat with EVT to reduced disability by at least one level on the mRS for one patient was 2.6 [35]. This benefit was confirmed across multiple subgroups (including patients older than 80 years and those with very severe strokes as indicated by a baseline NIHSS score greater than 20 [35]. Furthermore, the HERMES meta-analysis corroborated that the odds of better outcomes at 90 days with EVT declined with longer time from symptom onset to arterial puncture with each 1-h delay to reperfusion associated with a less favorable degree of disability (OR, 0.84 [95% CI, 0.76 to 0.93]; ARD, − 6.7%) and less functional independence (OR, 0.81 [95% CI, 0.71 to 0.92], ARD, − 5.2% [95% CI, − 8.3% to − 2.1%]) with benefit becoming non-significant after 7.3 h [36]. Based on the results of these successful clinical trials, EVT for AIS patients presenting within 6 h of symptom onset from LVO was recommended by the AHA/ASA as standard of care [5].

**Table 1:** Characteristics of the endovascular thrombectomy trials for anterior circulation acute ischemic stroke with large vessel occlusion

Study	Patient (*n*)	Key inclusion criteria	Key exclusion criteria	NIHSS, median	Main imaging modalities	Received IV tPA, %	EVT devices
MR CLEAN [8]	233	Age ≥ 18, NIHSS ≥ 2, LVO, IVT < 4.5 h, EVT < 6 h	BP > 185/110 mmHg, coagulopathy, active or recent hemorrhage	17	CT, CTA, CT perfusion (68%)	87	Retrievable stent
EXTEND-IA [9]	35	Age ≥ 18, NIHSS ≥ 6, LVO, IVT < 4.5 h, ischemic core <70 mL, mismatch volume ≥ 10 mL EVT < 6 h	Intracranial hemorrhage, any terminal illness	17	CT, CTA, CT perfusion	100	Solitaire device
ESCAPE [10]	165	Age ≥ 18, NIHSS ≥ 5, LVO, IVT < 4.5 h, small infarct core, EVT < 12 h	ASPECTS 0–5, no or minimal collaterals	16	CT, CTA	73	Available thrombectomy device
SWIFT PRIME [12]	98	Age 18–80, NIHSS 8–29, LVO, IVT < 4.5 h, small to moderate infarct core, EVT < 6 h	Hemorrhage, tumor, or vasculitis on CT or MRI, > 1/3 MCA territory or 100 mL infarct, DWI-ASPECTS ≤ 5	17	CT, CTA, CT perfusion	100	Solitaire stent retriever
REVASCAT [11]	103	Age 18–80, NIHSS ≥ 6, LVO, IVT < 4.5 h, EVT < 8 h	Large ischemic core (ASPECTS ≤ 7 on CT or 6 on DWI MRI)	17	CT, CTA, MRI	68	Solitaire stent retriever
THRACE [13]	414	Age 18–80, NIHSS 10–25, LVO, IVT < 4 h, EVT < 5 h	Cervical ICA stenosis/occlusion	18	CT, CTA, or MRA/MRI	100	Stent retriever, Penumbra
DAWN [14]	107	Age ≥ 18, NIHSS ≥ 10, LVO, small infarct core (< 1/3 MCA territory), a mismatch between clinical deficit and infarct volume EVT 6–24 h	Rapid improvement in neuro status, active or recent hemorrhage, coagulopathy	17	CT, CTA, MRA, CT perfusion, MR perfusion/diffusion	5	Trevo stent retriever
DEFUSE 3 [15]	92	Age 18–85, NIHSSS ≥ 6, LVO, ischemic core <70 mL, mismatch ratio > 1.8, mismatch volume ≥ 15 mL, or DWI volume < 25 mL EVT 6–16 h	BP > 185/110 mmHg, coagulopathy, ASPECTS score < 6 on non-contrast CT	16	CT perfusion 75%, MR perfusion/diffusion 25%	11	Any FDA-approved stent retriever

In modern clinical practice, combined techniques in which a direct aspiration first pass technique (ADAPT) is followed by a stent retrieval to remove any residual thrombus are commonly utilized especially for patients with long-segment occlusions and for intracranial ICA occlusions [37]. The most common technique is Solumbra, which derives its name from the simultaneous use of the Solitaire stent retriever and the Penumbra aspiration system. The technique has many variations using different stent retrieval devices as well as different guide catheters with or without a balloon guide catheter [37].

## Neuroimaging in Patient Selection for Endovascular Thrombectomy

There were great variabilities in the use of imaging tools for patient selection in the recent RCTs as shown in Table 1. In all trials, major early ischemic changes on baseline non-contrast CT (NCCT) were a reason for exclusion. NCCT and CTA were used to select patients with severe deficit and low infarct volume from LVO in most of the clinical trials [8, 9, 11–13]. Advanced imaging tools, including CT perfusion (CTP), diffusion/perfusion MRI, and MRA, were used to identify patients with perfusion mismatch (i.e., small infarct and large ischemic penumbra) in the EXTEND-IA and SWIFT PRIME trials [9, 12]. In the ESCAPE trial, multiphase CTA was used to evaluate the extent of collateral circulation, and patients with no or minimal collaterals were excluded from the study [10].

### Early Ischemic Changes on Imaging

The Alberta Stroke Program Early CT Score (ASPECTS) was originally developed to quantify early ischemic changes on NCCT and is a 10-point scoring system of anatomic regions distributed over the MCA territory on axial NCCT slices [38]. ASPECTS was utilized for patient selection in The ESCAPE, REVASCAT, and SWIFT PRIME trials and demonstrated to serve as a strong predictor of clinical outcome after EVT [11, 12, 35]. The MR CLEAN trial also utilized ASPECTS for patient screening; however, it did not use a threshold for patient exclusion [8]. The HERMES investigators’ meta-analysis of the pooled data from the MR CLEAN, ESCAPE, REVASCAT, SWIFT PRIME, and EXTEND-IA trials showed a clear benefit for thrombectomy in patients with ASPECT ≥ 6 [35]. When the treatment effect was analyzed for the 3 ASPECTS strata of 0–5, 6–8, and 9–10, there was a strong and consistent treatment effect for both ASPECTS 6–8 and 9–10 group with an adjusted odds ratio of 2.34 (95% CI: 1.68–3.26) and 2.66 (95% CI: 1.61–4.40), respectively [35]. There was no clear benefit for the 121 patients with ASPECT 0–5. These findings appear to have validated the use of ASPECTS score 6–10 as surrogate marker of small infarct volume [27].

### Imaging Modality to Screen for LVO

CTA of the head and neck is highly sensitive and specific for detection of LVO [39]. It also provides vasculature images on collaterals, aortic arch, vessel tortuosity, and cerebral ischemia [40–42]. CTA may help the interventionist to plan treatment strategy and reduce procedure time [41].

Reduced contrast enhancement on CTA source images suggests low cerebral blood volume (CBV) [35]. CTA source images appear to be more sensitive in predicting infarct volume and outcome than non-contrast CT [41, 43, 44]. Of note, slow contrast injection and quick image acquisition may cause overestimation of the infarct size. Most recent clinical trials used CTA to screen for LVO in patients with AIS [8–13].

Magnetic resonance angiography (MRA) is also a potential option for evaluation of LVO and collateral circulation [45, 46]. Time-of-flight (TOF) and contrast-enhanced (CE) MRA provide good vascular images through the neck and the circle of Willis [45]. CE MRA is performed with IV bolus of gadolinium. It is minimally invasive and offers better diagnostic accuracy than TOF-MRA in detecting LVO [45]. In the SWIFT PRIME, REVASCAT, and THRACE trials, MRA was used in select patients to screen for LVO [11–13].

### Assessment of Collateral Circulation

Collateral circulations are variable among patients [40, 47]. They were shown to predict the size of ischemic penumbra, infarct progression, and functional outcome after LVO [40, 47–49]. CTA, including multiphase or dynamic studies, is a very good imaging modality to assess collaterals [42, 43]. A major limitation of collateral assessment on CTA is that it is a single snap shot in time of contrast and may misdiagnose adequate collaterals as poor if the image is acquired early in the arterial phase [47, 49]. Digital subtraction angiography (DSA) remains the gold standard for triphasic evaluation of arterial, capillary, and venous circulation with high temporal and spatial resolution [41, 42]. The degree of leptomeningeal collaterals can be semi-quantified by comparing the retrograde pial arterial filling to the contralateral hemisphere [40, 49].

Optimal collateral circulation slows infarct progression and may be a good indication for EVT outside of the traditional time window [49, 50]. A good leptomeningeal collateral flow is associated with better functional outcome and lower rate of symptomatic intracranial hemorrhages after EVT [47, 48, 51, 52]. A large infarct core and poor collaterals are strong predictors of poor functional outcome [51, 52].

### Imaging Modality to Evaluate the Penumbra

CT perfusion (CTP) is a dynamic contrast-enhanced study developed for the evaluation of the infarct core and ischemic penumbra according to the estimated cerebral blood flow (CBF), mean transition time (MTT), and cerebral blood volume (CBV) [53–56]. The infarct core is defined as an area of brain tissue with > 70% reduction in CBF compared to the contralateral hemisphere, and the ischemic penumbra is defined as an area with > 6 s of delayed contrast arrival [41, 54–57]. The ischemic penumbra is identified by reduced CBF and normal CBV, whereas the infarct core has a matched decrease in both CBF and CBV [56–59]. The sizes of infarct core and ischemic penumbra are an indirect measurement of collaterals [60]. CTP is not reliable in patients with low cardiac output, cardiac arrhythmias, cervical internal carotid artery stenosis, or a combination of these conditions [61, 62].

CTP was performed only in 66.8% of the patients in the MR CLEAN trial [8]. Both EXTEND-IA and SWIFT PRIME used CTP to screen patients with small infarct core (IQR 4–32 mL and 0–16 mL, respectively) and large ischemic penumbra for EVT (Table 2) [9, 12]. Such strict selection criteria led to 60% and 71% favorable outcomes, respectively [9, 12], the highest ever reported with EVT. However, these studies may have excluded patients who could benefit from EVT [7, 13, 56, 58].

**Table 2:** Clinical infarct volume mismatch as eligibility criteria in recent landmark endovascular thrombectomy trials (adapted and modified by permission from Yu and Jiang [27])

	Median NIHSS (IQR)	Median ASPECTS (IQR)	Median infarct core per advanced imaging-mL (IQR)^*a*^	sICH^*b*^ (%)	Favorable outcome (%)
MR CLEAN [8]	17 (14–21)	9 (7–10)	–	7.7	33
EXTEND-IA [9]	17 (13–20)	NR	12 (4–32)	0	71
ESCAPE [10]	16 (13–20)	9 (8–10)	–	3.6	53
SWIFT PRIME [12]	17 (13–20)	9 (8–10)	6 (0–16)	1.0	60
REVASCAT [11]	17 (14–20)	7 (6–9)	–	1.9	44
THRAC [13]	18 (15–21)	5–10	–	2	53
DAWN [14]	17 (13–21)	NR	7.6 (2.0–18.0)	6	49
DEFUSE 3 [15]	16 (10–20)	8 (7–9)	9.4 (2.3–25.6)	7	45

*IQR* interquartile range, *NR* not reported

^a^Advanced imaging of perfusion CT or diffusion/perfusion MRI was used to quantify infarct core and ischemic penumbra [9, 12, 14, 15]

^b^sICH was defined as intraparenchymal hematoma, subarachnoid hemorrhage, or intraventricular hemorrhage associated with a worsening of the NIHSS score by ≥ 4 points within 24 h [63]

The diffusion/perfusion MRI is also very sensitive in the detection of infarct core and perfusion mismatch [56, 64–67]. MRI may predict clinical response to early reperfusion therapy [64, 65, 67–69]. However, tissue at risk can be overestimated by perfusion-weighted MRI [70].

Both CTP and MR perfusion can be performed with high-speed CT and MR imaging systems within 10 min [15, 56].

## Expanding the Therapeutic Time Window

Recently, the DAWN and DEFUSE 3 clinical trials completely disrupted the time window paradigm in AIS. In the DAWN trial, patients with a LVO AIS within 6 to 24 h of last known well were randomized to EVT vs standard of care alone [14]. The key inclusion criterion was the presence of a mismatch between the severity of clinical deficits and the volume of the ischemic core on MRI or CTP. The EVT arm and the control group contained 67% and 47%, respectively, of patients with stroke onset upon awakening. The 90-day rate of functional independence was 49% after EVT as compared to 13% in the control group, with patients treated with EVT at a median of 12.5 h from onset [14].

In the DEFUSE 3 trial, patients with a LVO AIS between 6 and 16 h after symptom onset were randomized to EVT vs standard of care alone [15]. The major inclusion criterion comprised the radiologic appearance of areas of mismatch between the ischemic core and the ischemic penumbra defined as an initial infarct volume < 70 mL, a ratio of ischemic penumbra to infarct core ≥ 1.8, and an absolute mismatch ≥ 15 mL. CTP was performed in 73% of the patients and diffusion/perfusion MRI was done in the other 27%. The EVT arm and the control group contained 49% and 42%, respectively, of patients with stroke onset upon awakening. The 90-day rate of functional independence was 45% after EVT as compared to 17% in the control group as well as an additional 20% absolute reduction in death or severe disability, with patients treated with EVT at a median of 11 h from onset [15].

Both DAWN and DEFUSE 3 trials demonstrated significant benefit of EVT within 16–24 h of last known well by selecting patients with clinical imaging mismatch (i.e., severe clinical deficit and small infarct core) per advanced imaging tools. The median NIHSS score with IQR was 17 (13–21) and 16 (10–20) while the median infarct core with IQR was 7.6 (2–18) and 9.4 (2.3–25.6) mL, respectively (Table 2) [14, 15]. The astoundingly large treatment effect in these late-window trials, termed the late-window paradox, has been attributed to both trials having enrolled patients with very slow infarct growth or progression rates [71]. These results led to a paradigm shift from “time window” to “tissue window” in the treatment of AIS based on utilization of advanced perfusion imaging. Accordingly, in response to these new data, the AHA/ASA guidelines recommend EVT under trial inclusion criteria for LVO AIS up to 24 h of last known well [5].

## Future Directions

### Access to Care

The advent of highly efficacious EVT for patients with LVO in the era of extended time window has created the need to revise acute stroke system of care. In the United States, typically there have been 3 designation levels of hospital certification in stroke management. These include Comprehensive Stroke Center (CSC), Primary Stroke Centers (PSC), and Acute Stroke Ready Hospital (ASRH), representing the highest to lowest level of stroke readiness. EVT for LVO is primarily offered in CSC [5]. Recently, a new level of care, Thrombectomy-Capable Stroke Centers (TSC), which falls between CSC and PSC has been established in light of the community need for greater access to thrombectomy [72]. However, the proper role of TSCs in the overall stroke system of care remains a controversy as some have raised the concern that inclusion of TSCs in urban areas with CSCs may lead to a lower quality of care by diluting volumes across the system [73, 74].

Furthermore, the benefit of patient diversion to different levels of stroke centers, including bypassing the closest PSC to go to a higher level of stroke care (TSC or CSC), remains uncertain [5, 75]. In a large prospective multicenter observational study with almost 1000 AIS patients with anterior circulation LVO, direct admission compared to interhospital transfer to an endovascular-capable stroke center (CSC or TSC) showed a faster median onset-to-revascularization time (202 vs 311.5 min; *p* < 0.001) with a higher proportion achieving functional independence (mRS≤ 2 at 90 days) (60% vs 52%; OR = 1.38; 95% CI, 1.06–1.79; *p* = 0.02) and excellent outcome (mRS 0–1 at 90 days) (47% vs 38%; OR = 1.47; 95% CI, 1.13–1.92; *p* = 0.005). Based on hypothetical bypass analysis, the authors estimated that limiting the bypass to the nearest endovascular-capable stroke center within 20 miles would result in only a 7-min delay in administration of IV r-tPA but would improve time to EVT by 94 min and accordingly improve outcomes [76]. In rural areas, however, as well as in urban areas with long travel times to CSCs, TSCs could reliably function in the region. Investigations are currently underway to address this challenge with novel modeling and prospective studies [77, 78].

A potential alternative approach proposed to meet the increasing demand for EVT in patients with AIS from LVO focuses on a mobile neuro-interventional team being dispatched to perform the EVT at the PSC rather than transferring the patient to the CSC. Two retrospective reviews based on stroke system of care in New York City and Hokkaido prefecture in Japan have demonstrated shorter door-to-puncture times in this “trip-and-treat” compared to the “drip-and-ship” models [79, 80]. The paradigm has recently been modeled in Germany and was a superior option to drip-and-ship transport with shorter door-to-puncture times [81, 82]. However, the door-to-puncture times in both “drip-and-drive” and “drip-and-ship” models were inferior to those of direct transfers [82].

### Patient Selection Criteria

#### Large Ischemic Stroke: Beyond Perfusion Imaging

In the most recent EVT clinical trials, patients with large baseline ischemic core lesion as measured by ASPECTS of < 6 or ischemic core volume > 70 mL were largely excluded from enrollment per protocol. Advanced perfusion imaging was used in 4 RCTs to define the best treatment effect of EVT [9, 12] and extend the treatment window up to 16–24 h of last known well [14, 15]. However, the median infarct core was only 12, 6, 7.6, and 9.4 mL in EXTEND-IA, SWIFT PRIME, DAWN, and DEFUSE 3 trials, [9, 12, 14, 15] respectively, (Table 2) as compared to 49.7 mL in MR CLEAN [8, 83]. Therefore, the best treatment effect is likely the results of strict selection of patients with small infarct core for EVT [9, 12, 14, 15]. There is growing amount of evidence highlighting the limitations of advanced imaging modalities in the real-world practice.

As suggested by the large treatment effect size observed in DAWN and DEFUSE 3 trials, the selection criteria based on the perfusion imaging thresholds were likely too stringent and could exclude a significant proportion of eligible patients. A number of recent studies have further corroborated this rationale by demonstrating that thrombectomy may benefit DAWN- and/or DEFUSE 3–ineligible patients. In a single-center study of 79 LVO AIS patients, comparison of admission infarct core per CTP and final infarct on follow-up CT showed that CTP overestimated infarct core by more than 10 mL in 38% of the patients [84]. In a matched case-controlled study of patients with LVO on CTA and baseline ischemic core greater than 50 mL on CTP, EVT was associated with significantly improved functional outcome at 90 days [85]. In a study of prospectively collected data, 38% of the DAWN-ineligible patients and 41% of DEFUSE 3–ineligible patients achieved functional independency at 90 days after EVT [86]. In another retrospective study, 30% of DAWN- and/or DEFUSE 3–ineligible patients achieved functional independence after off-label EVT [87]. Two additional studies showed that EVT could benefit patients with large infarct core (DWI-ASPECTS ≤ 5 or DWI lesion > 70 mL) [88, 89]. These suggestive signals of favorable outcomes for EVT in patients with large baseline core has led to several ongoing clinical trials including the TESLA (Thrombectomy for Emergent Salvage of Large Anterior Circulation Ischemic Stroke) trial (NCT03805308) and TENSION (Efficacy and Safety of Thrombectomy in Stroke With Extended Lesion and Extended Time Window) trial (NCT03094715). The TESLA trial will evaluate the effectiveness of EVT in patients with moderate-large infarcts (NCCT ASPECTS 2–5) at baseline, while the TENSION trial will investigate the effectiveness of EVT in patients with ASPECTS score of 3–5 and an extended time window (up to 12 h or unknown time of symptom onset).

Furthermore, the HERMES investigators demonstrated that a 30-min delay in imaging-to-reperfusion time had a similar adverse effect on the functional outcome as a 10-mL increase in ischemic core volume [90]. The HERMES meta-analysis also demonstrated that perfusion mismatch was not associated with either functional independence or functional improvement [35]. The use of perfusion imaging for patient selection has been shown as a potential cause of delay in reperfusion therapy [15], and in a recent cohort study, the use of advanced modality imaging was shown to delay EVT without improvement in clinical outcomes [91]. Therefore, perfusion-based patient selection may deny treatment to patients who might benefit from reperfusion therapy.

While effective clinical trial design in small sample size studies necessitates the use of advanced imaging tools for patient selection in order to achieve the best treatment effect, strict adherence to the perfusion imaging criteria of these RCTs may inadvertently deprive a significant proportion of patients in real-world practice from a proven therapy. Since 5 of the 8 RCTs that independently demonstrated the powerful efficacy of EVT validated the use of ASPECTS score for the assessment of early infarct [8, 10, 11, 13, 15], as shown in Table 2, a simple clinical deficit-CT imaging mismatch (i.e., high NIHSS score and ASPECTS) was proposed as selection criteria to guide EVT for all eligible patients in the fastest puncture-to-reperfusion time in the real-world practice [27].

#### Thrombectomy Beyond 24 Hours

The robust treatment effects of DAWN and DEFUSE 3 trials have been explained by the “late-window paradox,” postulating that slow progression of the ischemic core with sustained penumbra in AIS patients with LVO contributed to the efficacy of reperfusion therapy despite delayed initiation of treatment [71]. Further analysis of the control arm patients from the DEFUSE 3 trial demonstrated that approximately 20% of patients with an anterior circulation occlusion presenting within the extended time window and not treated with EVT continued to have a persistent favorable mismatch profile more than 38 h from their LKW time [92]. Furthermore, EVT was also reported to be safe and effective for patients who met all DAWN trial criteria but were treated beyond 24 h and up to 6 days of LKW time [93]. Most recently, in a single-centered retrospective review, AIS patients with anterior circulation LVOs treated with EVT beyond 16 h and up to 10 days of LKW time showed 11-fold higher odds of having an independent functional status at 3 months (mRS: 0–2) [94]. These recent data highlight the need for further clinical trials to determine if patients with a favorable perfusion profile would benefit from reperfusion treatment with EVT beyond 24 h.

#### “Mild” or “‘Non-disabling” Stroke

Since patients with “mild” or “non-disabling” stroke (NIHSS ***≤*** 5) were excluded from most of the successful EVT trials, the AHA/ASA guidelines do not recommend EVT of LVO in this subgroup [5]. However, recent literature has demonstrated that approximately 30% of AIS with LVO present with NIHSS ***≤*** 5. Consequently, most of these patients may be denied a proven therapy due to low NIHSS scores [95, 96]. Recently, retrospective studies have shown higher odds of improved outcomes for EVT in patients with LVO and NIHSS ***≤*** 5 [96–98]. Two multicenter clinical trials, ENDLOW (Endovascular Therapy for Low NIHSS Ischemic Strokes) trial (NCT04167527) in North America and MOSTE (Minor Stroke Therapy Evaluation) trial (NCT03796468) in Europe, are investigating the efficacy of EVT in patients with anterior circulation LVO and NIHSS ***≤*** 5 or 4, respectively.

### Device and Technology Development to Address Thrombus Characteristics

The properties of the thrombus are crucial elements in determining its responsiveness to EVT. The exact composition of a thrombus is related to its source and etiology but typically entails of fibrin and red blood cells (RBC) as well as minor white blood cells (WBC) content [99]. Hypodense, fibrin-rich thrombi show reduced recanalization rates regardless of technique [100]. Additionally, a fibrin-rich mature thrombus is firmer and less deformable in its interactions with the struts of a stent retriever device [101]. This reduced deformability accordingly increases friction between the thrombus and vessel wall, resulting in each pass at clot retrieval being less effective and potentially higher chance of EVT failure. Newer stent retriever designs that would exert increased radial force to capture the thrombus within their struts may be more effective in the removal of firmer fibrin-rich clots [102].

There are other distinct histological characteristics that may contribute to the understanding if certain thrombi are more resistant than others to extraction via EVT. Most notably, the amount of WBCs in a thrombus has also been associated with the facility of recanalization and duration of the procedure [103]. In more mature thrombi, a lot of WBCs have entered the thrombus, leading to its augmented organization and higher resilience to removal. The thrombus organization is closely related with its stability and its friction with the vessel wall, lending mature thrombi more difficult to extract [104]. Partial endothelialization at the edges of the thrombus [105] and thrombus of atypical origin such as calcified plaques [106], as well as the presence of other plasma constituents such as von Willebrand factor [107], are other features that have been reported as potential contributors associated with the development of mature and firmer thrombi more resistant to removal by EVT. Due to limited understanding of the thrombus histology by stroke neurologists and neuro-interventionalists, most patients with AIS from LVO are currently being treated in the same manner. Methods and technologies to determine the composition of the thrombi prior to EVT are a crucial translational research frontier that requires further investigation and undoubtedly will lead to more individualized treatment approaches rather than one size fits all.

### Rescue Therapy, Neuroprotection, and Other Adjuvant Treatments

Despite robust results from the recently successful EVT trials, more than 50% of patients in the EVT arm did not achieve good functional outcomes. The limitation of the reperfusion therapy stems from their reliance solely on early blood flow restoration without any other protection for the constituents of the neurovascular unit (NVU) in the brain [108, 109].

One of the possibilities for treatment failure is the target vessel re-occlusion. In patients with intracranial atherosclerosis–related LVO, there were reports of longer procedure time and higher rate of re-occlusion [110, 111]. Rescue therapy, including balloon angioplasty, stenting, and intra-arterial glycoprotein IIb/IIIa inhibitor infusion, was empirically used in some of those patients. The rescue therapy was shown to improve functional outcome without an increased risk of sICH. Randomized trials are warrantied to further investigate the safety and benefit of rescue therapy.

Although over 1000 agents targeting neuroprotection failed in clinical trials [108, 112], a few recent studies have demonstrated that some agents may have potential benefits as adjunct to reperfusion therapies. In a post hoc analysis of the URICO-ICTUS trial, administration of uric acid, an endogenous antioxidant, showed higher odds of achieving good functional outcome in subgroup of patients who received tPA and EVT than placebo [113]. In the phase II RHAPSODY trial, 3K3A-APC, a recombinant variant of human activated protein C (APC), in combination with thrombolysis and EVT, showed a trend towards reduced hemorrhage [114]. In a recent multicenter ESCAPE NA1 trial, Nerinetide, an excitotoxic cell death pathway inhibitor, failed to improve a 90-day functional outcome in patients undergoing EVT plus medical therapy. However, the outcome was improved in patients who did not receive tPA [115]. Hence, neuroprotective agents remain a potential adjuvant therapy and are warrantied for further investigation in AIS intervention.

## Conclusions

The past 5 years has witnessed a revolution in cerebral recanalization therapy for patients with acute LVO. However, there remain many unanswered questions for further investigation, whereby this proven therapy can be expeditiously, safely, and judiciously provided to all eligible patients whom may benefit. Over the next decade, various endeavors will expound on optimization and modifications of stroke systems of care, imaging modality, and clinical criteria to enhance patient selection, as well as technological advancements, rescue, and adjuvant therapies to deliver more efficacious treatment.

## Appendix of Clinical Trials

**Table:**

ENDLOW	Endovascular Therapy for Low NIHSS Ischemic Strokes
ESCAPE	Endovascular Treatment for Small Core and Anterior Circulation Proximal Occlusion With Emphasis on Minimizing CT to Recanalization Times
ESCAPE-NA1	Safety and Efficacy of NA-1 in Subjects Undergoing Endovascular Thrombectomy for Stroke
EXTEND-IA	Extending the Time for Thrombolysis in Emergency Neurological Deficits – Intra-Arterial
IMS III	Interventional Management of Stroke III
MERCI	Mechanical Embolus Removal in Cerebral Ischemia
MR CLEAN	Multicenter Randomized Clinical Trial of Endovascular Treatment for Acute Ischemic Stroke in the Netherlands
MR RESCUE	Mechanical Retrieval and Recanalization of Stroke Clots Using Embolectomy
PROACT-II	Prolyse in Acute Cerebral Thromboembolism II
REVASCAT	Randomized trial of revascularization with Solitaire FR device versus best medical therapy in the treatment of acute stroke due to anterior circulation large vessel occlusion presenting within 8 h of symptom onset
RHAPSODY	Safety evaluation of 3K3A-APC in ischemic stroke
SWIFT	SOLITAIRE™ With the Intention for Thrombectomy
SWIFT PRIME	Solitaire With the Intention for Thrombectomy as Primary Endovascular Treatment
SYNTEHSIS Expansion	Intra-Arterial Versus Systemic Thrombolysis for Acute Ischemic Stroke
TESLA	Thrombectomy for Emergent Salvage of Large Anterior Circulation Ischemic Stroke
THERAPY	The Randomized, Concurrent Controlled Trial to Assess the Penumbra System’s Safety and Effectiveness in the Treatment of Acute Stroke
THRACE	Trial and Cost Effectiveness of Intra-arterial Thrombectomy in Acute Ischemic Stroke
TREVO 2	Thrombectomy Revascularization of Large Vessel Occlusions in Acute Ischemic Stroke
URICO-ICTUS	Safety and efficacy of uric acid in patients with acute stroke
